# Addressing the burden and associated factors of undernutrition in under-five children with congenital heart disease in Pakistan: evidence from a multicenter cross-sectional study

**DOI:** 10.3389/fnut.2026.1953133

**Published:** 2026-10-16

**Authors:** Muhammad Imran Khan, Ijaz ul Haq, Muhammad Awais, Shahbaz Ahmad Zakki, Sohail Akhtar, Muhammad Ayaz Ghulam Nabi, Elmuez Alsir Ahmed Aboagarib, Manjur Kolhar, Xianghui Huang, Xiaojing Hu

**Affiliations:** 1 Fujian Key Laboratory of Neonatal Diseases, Xiamen, Fujian, China; 2 Xiamen Children's Hospital (Children's Hospital of Fudan University at Xiamen), Xiamen, Fujian, China; 3 Department of Public Health and Nutrition, The University of Haripur, Haripur, Pakistan; 4 Department of Clinical Nutrition, College of Applied Medical Sciences, King Faisal University, Al-Ahsa, Saudi Arabia; 5 Department of Health Information Management and Technology, College of Applied Medical Sciences, King Faisal University, Al-Ahsa, Saudi Arabia; 6 Pediatrics Department, College of Medicine, King Faisal University, Al-Ahsa, Saudi Arabia; 7 Children's Hospital of Fudan University, Shanghai, China; 8 National Management Office of Neonatal Screening Project for Congenital Heart Disease (CHD), National Children's Medical Center, Children's Hospital of Fudan University, Shanghai, China

**Keywords:** congenital heart disease, nutritional status, under five children, undernutrition, ventricular septal defect

## Abstract

**Background:**

Children with congenital heart disease (CHD) are at heightened risk of undernutrition due to elevated metabolic demands, feeding intolerance, and growth failure. This study aimed to identify the associated factors of undernutrition among children under 5 years with CHD in Pakistan.

**Methods:**

A hospital-based study was carried out among 197 confirmed CHD across three tertiary hospitals in Bannu, Khyber Pakhtunkhwa (KP). WHO-recommended standards and structured questionnaires were used. Multivariable logistic regression analysis was performed to identify independent predictors of undernutrition.

**Results:**

Overall, 58.9% were male, 40.1% were stunted, 44.2% were underweight, and 35.5% were wasted. Ventricular septal defect was the most common CHD (34.5%), followed by patent ductus arteriosus (23.4%). Infants aged 6–11 months had the most severe deficits. Male children had significantly higher odds of wasting than female children (adjusted odds ratio [aOR] = 2.10, 95% CI: 1.10–3.98), whereas children from households with a monthly income of ≥ PKR 40,000 had lower odds of wasting (aOR = 0.45, 95% CI: 0.24–0.84) and underweight (aOR = 0.52, 95% CI: 0.29–0.94). Similarly, children whose fathers were government employees (aOR = 0.30, 95% CI: 0.09–0.98) also had lower odds of being underweight. No sociodemographic or clinical variables were significantly associated with stunting (all *p* > 0.05).

**Conclusion:**

Undernutrition exhibits a high distribution and severity among young CHD children. Multiple associated factors of undernutrition based on wasting and underweight were identified. Timely referrals for nutritional support are of great significance in preventing nutritional deterioration.

## Introduction

CHD refers to structural abnormalities of the heart or great vessels present at birth (1). Among the more than 25 recognized types, septal defects, encompassing atrial septal defect (ASD), ventricular septal defect (VSD), and atrioventricular septal defect (AVSD), constitute approximately 40% of the cases (2). CHD is the most common congenital anomaly globally, affecting approximately 1.5 million newborns annually (3). In 2010–2017, it caused 9.4 infant deaths per 1,000 live births in Pakistan, ranking seventh among causes of infant mortality (4). Children with CHD face markedly elevated risk of undernutrition due to pathophysiological drivers: increased metabolic demands, feeding intolerance, impaired nutrient absorption, fluid restrictions, heart failure, and chronic hypoxia-induced polycythemia. These factors collectively impair growth, weaken myocardial and respiratory muscle function, compromise immunity, and increase susceptibility to infection, contributing to higher hospitalization rates, poorer surgical outcomes, stunted development, and excess mortality (5).

A tertiary hospital in Karachi reported that approximately 60,000 infants were born annually with congenital malformations. Among them, 60% experience early-stage mortality, which indicated a relatively high mortality rate among patients with CHD (6). In research conducted in Faisalabad, ASD emerged as the most observed cardiac defect, accounting for 41.5% of the cases. This was followed by tetralogy of Fallot (TOF) at 21%, VSD at 14%, and patent ductus arteriosus (PDA) at 8% (7). In Khyber Pakhtunkhwa (KP), the regional distribution of CHD prevalence indicated the highest rates in the Peshawar division, reaching 36.98%. Subsequently, the Malakand division had a prevalence rate of 28.84%, the Mardan division 16.71%, the Kohat division 6.1%, the Bannu division 4.3%, the Hazara division 3.5%, and the Dera Ismail Khan division 3.5% (8).

According to the World Health Organization (WHO), approximately 15%−64% of children suffering from CHD experience undernutrition (9). Although population-wide data are scarce in developing nations such as Pakistan, where hospital-based investigations consistently indicate high incidences of malnutrition among children suffering from CHD (10). Data obtained from the Pakistan Institute of Cardiology (PIC) in Peshawar indicated that the overall prevalence of malnutrition among children with CHD was 86.7%. Specifically, 13.3% of the children were free from malnutrition, 27% exhibited mild malnutrition, 36.6% had moderate malnutrition, and 23% suffered from severe malnutrition (11). Generally, children in areas with high poverty and limited resources have a higher risk of undernutrition, wasting, and stunting. Multiple factors contribute to undernutrition among children with CHD, such as hemodynamic instability, reduced nutritional intake resulting from feeding fatigue, impaired food absorption, fluid restriction, heart failure, polycythemia caused by persistent hypoxia, and increased metabolic requirements due to the pathophysiology of CHD (12). Furthermore, the cardiac and respiratory muscles are impacted, entailing the atrophy of muscle mass, which results in impaired myocardial and respiratory functions. Moreover, immunological competence and reparative capacity are influenced, increasing the susceptibility to infections (4).

In the context of this concurrent epidemic of cardiac and nutritional vulnerability, especially among young children in resource-constrained settings, early diagnosis alone is inadequate. An effective response requires integrated clinical measures, including routine anthropometric screening at initial contact for CHD, family-centered feeding counseling, and systematic linkage of cardiac services with community growth monitoring and social support. Therefore, this study evaluated the nutritional status and identified the modifiable predictors of undernutrition among children under 5 years of age with echocardiographically confirmed CHD across three tertiary hospitals in Bannu District, KP, Pakistan.

## Materials and methods

### Study setting, design, and duration

This multicenter cross-sectional study was conducted from December 2024 to May 2025 across three tertiary care hospitals in Bannu District, Khyber Pakhtunkhwa, Pakistan: Khalifa Gul Nawaz Teaching Hospital; District Headquarter Teaching Hospital; and Women and Children Teaching Hospital. These hospitals are affiliated with the Medical Teaching Institution (MTI) Bannu. Participants were children aged 6–59 months with echocardiographically confirmed CHD, recruited consecutively from pediatric and cardiology departments.

### Sample size

The required sample size was calculated using the standard formula for estimating a single population proportion: *n* = (*Z*^2^ × *p* × *q*) / *e*^2^, where *Z* = 1.96 (for 95% confidence level), *p* = 0.043 (estimated CHD prevalence), *q* = 1 – *p*, and *e* = 0.03 (3% margin of error). After accounting for a 10% non-response rate, the final target sample size was 196 children aged 6–59 months with echocardiographically confirmed CHD and their primary caregivers who completed valid sociodemographic and feeding practice questionnaires. Finally, 197 respondents were included in the final analysis.

### Exclusion criteria

Children with co-occurring major congenital anomalies (e.g., cleft lip or palate), chromosomal or monogenic disorders (e.g., Down syndrome, Turner syndrome, DiGeorge syndrome), or chronic medical conditions known to independently impair growth or nutrient absorption (e.g., chronic kidney disease, celiac disease, cystic fibrosis, short bowel syndrome, lactose intolerance) were excluded.

### Data collection procedure

Data were collected by pediatric residents and cardiology fellows, who were trained by the Principal Investigator on standardized anthropometric measurement techniques, structured questionnaire administration, and ethical protocols for working with CHD children. All measurements were performed by trained medical doctors under direct supervision; caregivers provided written informed consent prior to any data collection. Age of the participants was confirmed from the vaccination card or Form B issued by NADRA of Pakistan. Sociodemographic and feeding practice data were collected in participants' preferred language (Pashto or Urdu) using validated, pretested questionnaires. CHD was classified based on echocardiographic findings into anatomical subtypes (ASD, VSD, PDA, TOF, and TGA) and further stratified as cyanotic or acyanotic (13). CHD cases were confirmed and classified by a pediatric cardiologist. Complete data set was obtained without missing a single individual or observation.

### Anthropometric measurements

All eligible children underwent standardized anthropometric assessment using calibrated equipment: digital weighing scales (to the nearest 0.1 kg) and infant length boards or standing stadiometers (to the nearest 0.1 cm), following WHO-recommended protocols. Anthropometric measurements were recorded twice, and the average value was documented on the data collection sheet. Length was measured for children < 2 years; height was measured for those ≥2 years. Nutritional status was classified using WHO 2006 Child Growth Standards: Stunting (height-for-age *Z*-score [HAZ] < −2), wasting (weight-for-height *Z*-score [WHZ] < −2), and underweight (weight-for-age *Z*-score [WAZ] < −2) (12).

### Data analysis

WHO Anthro software was used to calculate the anthropometric z-scores, and IBM SPSS Statistics version 22.0 was used for further statistical analysis. There was no missing data. Descriptive statistics (frequencies, percentages, means ± SDs) were computed to summarize sociodemographic, clinical, and anthropometric variables. Multivariate logistic regression was performed to screen potential associated factors of stunting, wasting, and underweight to identify independent associations, with results reported as adjusted odds ratios (aORs) and 95% confidence intervals. The Hosmer–Lemeshow test was used for goodness of model fit, and the variance inflation factor (VIF) was used for multicollinearity among independent variables, with VIF values < 5 indicating the absence of significant multicollinearity. Statistical significance was defined as *p* < 0.05 (two-tailed).

## Results

### Socio-demographic characteristics of the study population

The respondents' sociodemographic characteristics are shown in Table 1. The cohort was male-predominant (58.9%), predominantly rural-dwelling (80.7%), and had a mean age of 23.1 months (SD = 16.8). Most children were born in healthcare facilities (68.5%); fathers were primarily engaged in manual labor or agriculture (54.3%), and mothers were overwhelmingly homemakers (98.5%). Parental educational attainment was low: 68.5% of fathers and 82.7% of mothers had completed no formal schooling or only primary education.

**Table 1:** Socio-demographic characteristics of participants (*n* = 197).

Variable	Category	Frequency, *n*	Mean ±SD or (%)
Child's gender	Male	116	58.9
	Female	81	41.1
Child's age (in months)		–	23.1 ± 16.8
Mother's age (years)		–	28.14 ± 5.6
Household monthly income (PKR)		–	43015.2 ± 10479.7
Birth location	Healthcare facility	135	68.5
	Home	62	31.5
Birth spacing Interval (years)	< 2	57	28.9
	Firstborn	27	13.7
	≥ 2	113	57.4
Birth order	First	32	16.2
	Second	53	26.9
	Third	52	26.4
	Fourth or higher	60	30.5
Father's occupation	Manual labor/agriculture	107	54.3
	Government employee	19	9.6
	Private sector employee	33	16.8
	Self-employed	26	13.1
	Driver	12	6.1
Mother's occupation	Homemaker	194	98.5
	Government employee	3	1.5
Father's highest education	No formal schooling	111	56.3
	Primary school	24	12.2
	Secondary school	42	21.3
	Higher education	20	10.2
Mother's highest education	No formal schooling	142	72.1
	Primary school	21	10.6
	Secondary school	20	10.2
	Higher education	14	7.1
Residence	Rural	159	80.7
	Urban	38	19.3

### Clinical characteristics of children with CHD

Among the 197 children with echocardiographically confirmed CHD, VSD was the most prevalent lesion (34.5%), followed by PDA (23.4%), ASD (21.3%), TOF (14.2%), transposition of the great arteries (TGA; 5.1%), and coarctation of the aorta (COA; 1.5%) (Supplementary Table 1). The vast majority had no first- or second-degree family history of CHD (95.9%). At the time of assessment, 68.0% were receiving cardiac-specific pharmacotherapy (e.g., diuretics, beta-blockers, or digoxin) and 20.3% had undergone prior corrective or palliative surgery or catheter-based intervention.

### Nutritional status of children with CHD

Anthropometric assessment revealed a mean weight of 9.71 kg (SD = 3.34), mean length (for children < 24 months) or height (for children ≥24 months) of 78.02 cm (SD = 14.32), and mean mid-upper arm circumference (MUAC) of 12.01 cm (SD = 1.89) (Supplementary Table 2).

WHO growth standard-based *Z*-score analysis revealed a high burden of malnutrition (Figures 1A, B). The mean *Z*-scores were notably below the reference median for HAZ (−1.82, 95% CI: −1.95 to −1.69), WAZ (−1.84, 95% CI: −1.98 to −1.71), and WHZ (−1.09, 95% CI: −1.23 to −0.95), indicating moderate-to-severe undernutrition. The youngest age group (6–11 months) exhibited the most severe deficits, with mean WAZ (−2.46) and HAZ (−2.08) falling below the −2 threshold, while older children (25–59 months) showed relatively better, though still suboptimal growth parameters. Boys demonstrated worse stunting (HAZ: −1.93 vs. −1.71), whereas girls showed slightly higher wasting (WHZ: −1.14 vs. −1.05). The data has been shown in Supplementary Table 1.

**Figure 1:**
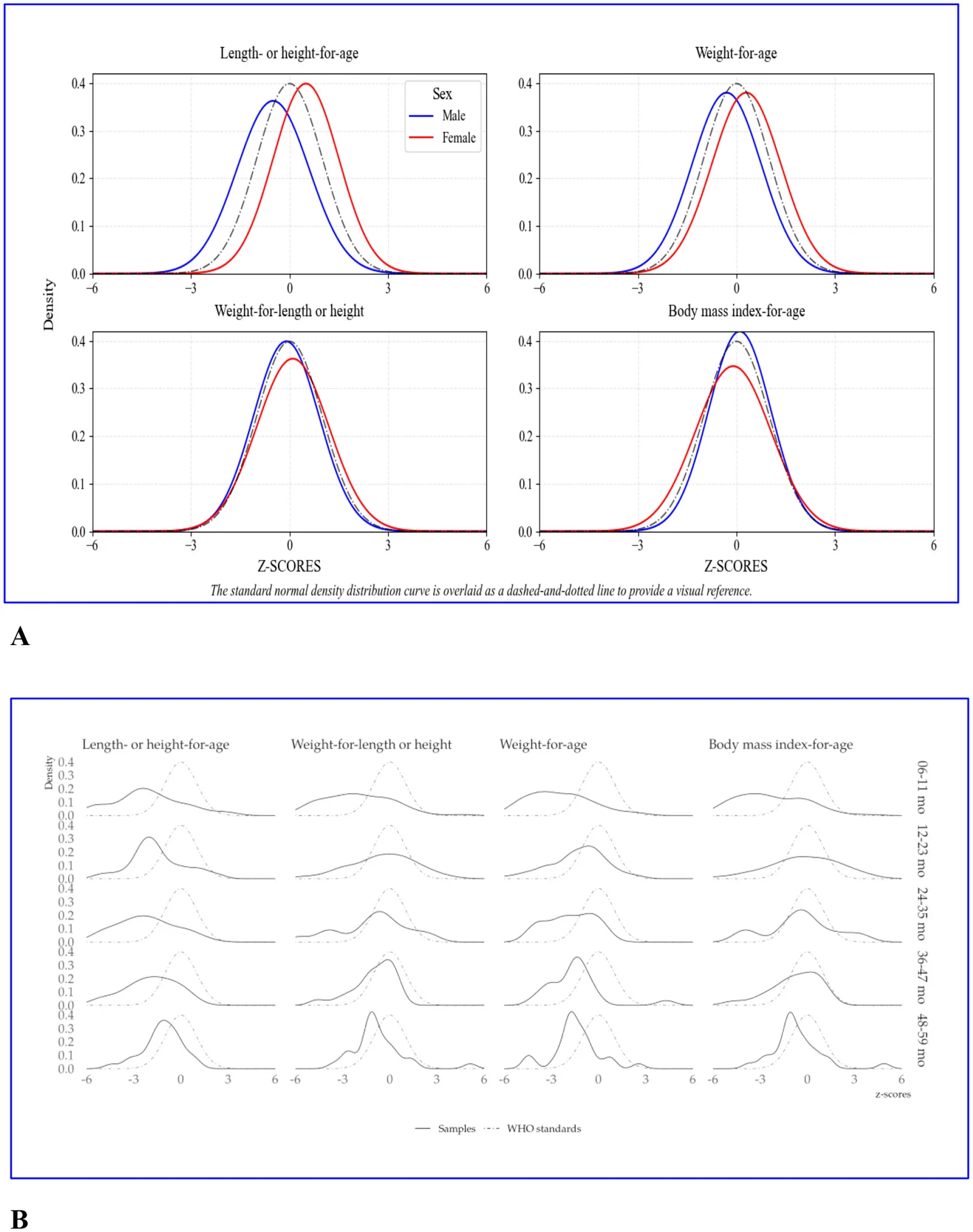
**(A)** *Z*-score distribution stratified according to age group and gender among children diagnosed with CHD. **(B)** *Z*-score distribution stratified by age group and gender among children with CHD.

The distribution of undernutrition was high. Overall, 44.2% of the children were underweight, 40.1% were stunted, and 35.5% suffered from wasting (Supplementary Figure 1). These findings indicate a substantial burden of both chronic (stunting) and acute (underweight and wasting) undernutrition among children with CHD.

### Associated factors of wasting among children with CHD

Table 2 presents the sociodemographic and clinical factors associated with wasting. Male children had significantly higher odds of wasting compared with female children (aOR = 2.10; 95% CI: 1.10–3.98; *p* = 0.02). Children from households with a monthly income of ≥ PKR 40,000 had significantly lower odds of wasting than those from lower-income households (aOR = 0.45; 95% CI: 0.24–0.84; *p* = 0.01), indicating a protective effect of higher household income.

**Table 2:** Sociodemographic and clinical factors associated with wasting in children with CHD.

Variable	Category	aOR (95% CI)	*p*-value
Gender	Male	2.1 (1.10–3.98)	0.02^*^
	Female	–	–
Child's age (months)	6–24 months	Ref	–
	25–60 months	0.82 (0.43–1.56)	0.55
Sibling with CHD	Yes	1.7 (0.35–8.10)	0.52
	No	–	–
Locality	Urban	0.65 (0.28–1.50)	0.31
	Rural	–	–
Birth spacing (years)	< 2	Ref	–
	First child	0.71 (0.25–2.01)	0.52
	≥2	0.89 (0.46–1.72)	0.73
Birth order	1st	Ref	–
	2nd	1.28 (0.50–3.28)	0.61
	3rd	1.55 (0.60–4.00)	0.36
	≥4th	0.97 (0.37–2.54)	0.95
Father's education	No formal education	Ref	–
	Primary	0.53 (0.19–1.45)	0.22
	Secondary	0.88 (0.41–1.89)	0.75
	Higher	0.48 (0.16–1.46)	0.19
Mother's education	No formal education	Ref	–
	Primary	0.82 (0.30–2.22)	0.7
	Secondary	0.65 (0.23–1.85)	0.42
	Higher	0.28 (0.06–1.35)	0.11
Household income (PKR)	< 40	Ref	–
	≥40	0.45 (0.24–0.84)	0.01^*^
Birthplace	Hospital	Ref	–
	Home	1.30 (0.68–2.48)	0.42
Father's occupation	Farmers/Labor	Ref	–
	Government employee	0.47 (0.15–1.48)	0.2
	Private sector employee	0.76 (0.33–1.75)	0.52
	Self-employed	0.53 (0.20–1.40)	0.2
	Driver	0.14 (0.02–1.08)	0.06
Mother's occupation	Housewife	Ref	–
	Government employee	0.89 (0.08–10.20)	0.92
VSD	Yes	1.02 (0.58–1.80)	0.95
	No	–	–
ASD	Yes	0.68 (0.38–1.22)	0.2
	No	–	–
PDA	Yes	0.58 (0.31–1.08)	0.09
	No	–	–
TOF	Yes	0.92 (0.40–2.12)	0.85
	No	–	–
CHD-specific pharmacotherapy	Yes	0.65 (0.33–1.28)	0.21
	No	–	–
Caregiver chronic illness	Yes	1.65 (0.10–27.20)	0.73
	No	–	–

Significant level: ^*^p < 0.05 is significant.

Among the clinical variables, no statistically significant associations were observed for ventricular septal defect (VSD), atrial septal defect (ASD), patent ductus arteriosus (PDA), tetralogy of Fallot (TOF), or receipt of CHD-specific pharmacotherapy (all *p* > 0.05). Similarly, no statistically significant associations were found for child age, sibling history of CHD, locality, birth spacing, birth order, parental education, birthplace, parental occupation, maternal occupation, or caregiver chronic illness (all *p* > 0.05).

### Associated factors of stunting among children with CHD

Table 3 presents the sociodemographic and clinical factors associated with stunting among children with CHD. After adjustment for potential confounding variables, no sociodemographic or clinical factor was found to be significantly associated with stunting (all *p* > 0.05).

**Table 3:** Sociodemographic and clinical factors associated with stunting in children with CHD.

Variable	Category	aOR (95% CI)	*p*-value
Gender	Male	0.92 (0.50–1.69)	0.79
	Female	–	–
Child's Age (months)	6–24 months	Ref	–
	25–60 months	0.74 (0.40–1.37)	0.34
Sibling with CHD	Yes	1.15 (0.22–6.02)	0.87
	No	–	–
Locality	Urban	0.68 (0.29–1.58)	0.37
	Rural	–	–
Birth spacing (years)	< 2	Ref	–
	First child	0.68 (0.26–1.78)	0.43
	≥2	0.85 (0.44–1.64)	0.63
Birth order	1st	Ref	–
	2^nd^	0.92 (0.38–2.23)	0.85
	3^rd^	1.08 (0.44–2.65)	0.87
	≥4th	0.96 (0.39–2.36)	0.93
Father's education	No formal education	Ref	–
	Primary	0.88 (0.35–2.21)	0.78
	Secondary	0.71 (0.33–1.53)	0.38
	Higher	0.85 (0.31–2.33)	0.75
Mother's education	No formal education	Ref	–
	Primary	1.08 (0.42–2.78)	0.87
	Secondary	0.56 (0.20–1.57)	0.27
	Higher	0.42 (0.12–1.47)	0.17
Household income (PKR)	< 40	Ref	–
	≥40	0.65 (0.36–1.17)	0.15
Birthplace	Hospital	Ref	–
	Home	1.42 (0.74–2.72)	0.29
Father's occupation	Farmers/Labor	Ref	–
	Government employee	0.62 (0.22–1.74)	0.37
	Private sector employee	0.67 (0.30–1.50)	0.33
	Self-employed	0.57 (0.23–1.42)	0.23
	Driver	0.31 (0.07–1.34)	0.12
Mother's occupation	Housewife	Ref	–
	Government employee	2.28 (0.20–26.0)	0.51
VSD	Yes	0.78 (0.43–1.42)	0.42
	No	–	–
ASD	Yes	0.62 (0.44–1.51)	0.11
	No	–	–
PDA	Yes	1.58 (0.82–3.05)	0.17
	No	–	–
TOF	Yes	1.75 (0.76–4.02)	0.19
	No	–	–
CHD-specific pharmacotherapy	Yes	1.18 (0.64–2.18)	0.6
	No	–	–
Caregiver chronic illness	Yes	1.12 (0.07–18.80)	0.94
	No	–	–

Although some variables demonstrated trends toward an association, including atrial septal defect (ASD) (aOR = 0.62; 95% CI: 0.44–1.51; *p* = 0.11), patent ductus arteriosus (PDA) (aOR = 1.58; 95% CI: 0.82–3.05; *p* = 0.17), tetralogy of Fallot (TOF) (aOR = 1.75; 95% CI: 0.76–4.02; *p* = 0.19), and household income ≥ PKR 40,000 (aOR = 0.65; 95% CI: 0.36–1.17; *p* = 0.15), none reached statistical significance. Likewise, child sex, age, sibling history of CHD, locality, birth spacing, birth order, parental education, birthplace, parental occupation, maternal occupation, ventricular septal defect (VSD), CHD-specific pharmacotherapy, and caregiver chronic illness were not significantly associated with stunting (all *p* > 0.05).

### Associated factors of underweight among children with CHD

Table 4 presents the sociodemographic and clinical factors associated with underweight among children with CHD. Children from households with a monthly income of ≥ PKR 40,000 had significantly lower odds of being underweight than those from households with lower income (aOR = 0.52; 95% CI: 0.29–0.94; *p* = 0.03). In addition, children whose fathers were government employees had significantly lower odds of underweight than those whose fathers were farmers or laborers (aOR = 0.30; 95% CI: 0.09–0.98; *p* = 0.04).

**Table 4:** Sociodemographic and clinical factors associated with underweight in children with CHD.

Variable	Category	aOR (95% CI)	*p*-value
Gender	Male	1.38 (0.76–2.51)	0.29
	Female	–	–
Child's age (months)	6–24 months	Ref	–
	25–60 months	1.05 (0.57–1.93)	0.87
Sibling with CHD	Yes	2.35 (0.53–10.40)	0.26
	No	–	–
Locality	Urban	0.48 (0.20–1.15)	0.1
	Rural	–	–
Birth spacing (years)	< 2	Ref	–
	First child	0.45 (0.16–1.26)	0.13
	≥2	0.73 (0.38–1.40)	0.34
Birth order	1st	Ref	–
	2nd	0.85 (0.34–2.13)	0.73
	3rd	1.20 (0.47–3.06)	0.7
	≥4th	0.98 (0.39–2.46)	0.97
Father's education	No formal education	Ref	–
	Primary	0.55 (0.21–1.44)	0.22
	Secondary	0.55 (0.25–1.20)	0.13
	Higher	0.47 (0.16–1.38)	0.17
Mother's education	No formal education	Ref	–
	Primary	0.92 (0.36–2.35)	0.86
	Secondary	0.49 (0.17–1.41)	0.18
	Higher	0.22 (0.05–1.01)	0.05
Household income (PKR)	< 40	Ref	–
	≥40	0.52 (0.29–0.94)	0.03^*^
Birthplace	Hospital	Ref	–
	Home	0.65 (0.31–1.21)	0.08
Father's Occupation	Farmers/Labor	Ref	–
	Government employee	0.30 (0.09–0.98)	0.04^*^
	Private sector employee	0.71 (0.31–1.63)	0.42
	Self-employed	0.68 (0.27–1.71)	0.41
	Driver	0.23 (0.05–1.12)	0.07
Mother's Occupation	Housewife	Ref	–
	Government employee	2.78 (0.24–32.2)	0.42
VSD	Yes	0.82 (0.44–1.53)	0.53
	No	–	–
ASD	Yes	0.85 (0.44–1.64)	0.63
	No	–	–
PDA	Yes	0.72 (0.36–1.44)	0.35
	No	–	–
TOF	Yes	0.75 (0.34–1.65)	0.48
	No	–	–
CHD-specific pharmacotherapy	Yes	0.92 (0.48–1.76)	0.8
	No	–	–
Caregiver chronic illness	Yes	1.38 (0.08–23.50)	0.82
	No	–	–

aOR, adjusted odds ratio; Ref, reference category; CI, confidence interval. Significance levels: ^*^p < 0.05.

No statistically significant associations were observed between underweight and child sex, age, sibling history of CHD, locality, birth spacing, birth order, parental education, maternal occupation, ventricular septal defect (VSD), atrial septal defect (ASD), patent ductus arteriosus (PDA), tetralogy of Fallot (TOF), receipt of CHD-specific pharmacotherapy, or caregiver chronic illness (all *p* > 0.05). Although higher maternal education was associated with a lower odds of underweight, the association was of borderline statistical significance (aOR = 0.22; 95% CI: 0.05–1.01; *p* = 0.05).

## Discussion

The current study demonstrated a high distribution of undernutrition among CHD children. Specifically, 40.1 % of the children experienced stunting, 44.2% were underweight, and 35.5 % suffered from wasting, suggesting the concurrent presence of chronic and acute nutritional deficiencies. Regarding the distribution of CHD, VSD was more prevalent, followed by PDA and ASD. Crucially, infants aged 6–11 months demonstrated the most severe anthropometric deficits, underscoring this developmental window as a high-priority target for integrated nutritional and cardiac care. Older children (25–59 months) showed relatively better nutritional status as compared to other groups. Multiple sociodemographic risk factors of undernutrition have been identified. This research provides regionally specific evidence indicating that malnutrition presented in the form of stunting, underweight, and wasting impacts nearly half of the children diagnosed with CHD. Undernutrition is highly prevalent among young children, with the greatest nutritional deficits observed during infancy (6–11 months). Wasting was independently associated with male sex, whereas higher household income was protective against both wasting and underweight. Fathers employed in the government sector were also associated with lower odds of underweight, while no sociodemographic or clinical factor was significantly associated with stunting after adjustment. The distribution of underweight, wasting and stunting among CHD children was high in our study as compared to the findings of the Pakistan Health and Demographic Survey, which reported 38 % stunting, 23 % underweight and 7 % wasting (14). This might be because CHD triggers undernutrition parameters due to increased metabolic demands and feeding challenges (15). A previous study in Indonesia reported that 563 (49.0%) children with CHD were underweight, 549 (47.8%) were stunted, and 361 (31.4%) were wasted (16). The prevalence of stunting (40.1%) exceeds rates reported in high-income countries (21%−24%) and is close to findings from Uganda (45%), affirming the disproportionate impact of CHD on linear growth in LMICs (17, 18). Underweight (44.2%) further indicates a concurrent acute and chronic energy deficit, which is aggravated by CHD-associated feeding challenges, such as dysphagia, fatigue during feeding, and gastroesophageal reflux, as well as an increased infection burden (19).

Sociodemographic factors demonstrated robust, graded associations. Specifically, government employment and household income equal to or greater than PKR 40,000 each provided significant protection against underweight. Previously, it has been documented that parents' employment and family income are independent risk factors for underweight CHD children (4). These associations not only mirror material benefits but also heightened health literacy, care coordination, and treatment compliance, which consistently correlated with improved outcomes in pediatric CHD (4). These findings may reflect broader socioeconomic and healthcare-access disparities; however, potential mechanisms such as health literacy and treatment adherence were not directly assessed in the current study.

The current study did not identify any significant factors associated with stunting among children with CHD. The lack of statistical significance does not necessarily indicate the absence of a clinically meaningful association. Possible explanations include limited sample size, insufficient variability in relevant exposures, heterogeneity in CHD severity, unmeasured clinical confounding, measurement-related factors, and the cross-sectional nature of the study.

Wasting was observed in 35.5 % of the children, which was significantly correlated with monthly income and male gender, consistent with the broader associations between sociodemographic deprivation and acute malnutrition. Our finding indicating that the male sex was associated with 2.1-fold higher odds of wasting, which is consistent with previous evidence from India reporting an increased incidence of wasting among boys with CHD. Males had 1.22 times more odds than female of having CHD (15). An Indian study reported that low socioeconomic scale was an independent risk factors of malnutrition in univariate analysis (20). A study in Africa concluded that malnutrition is common among CHD children in low-income settings (12). The situation might be the same for CHD children residing in high income countries as well (5). This situation is particularly worrisome as CHD is associated with elevated metabolic requirements, difficulties in feeding, and suboptimal nutrient absorption (20). These factors suggest insufficient nutrient intake and chronic exposure to infections, along with substandard care practices during the crucial early stages of life. Consequently, there is an urgent requirement for personalized nutritional interventions starting from infancy (21). There exist specific factors influencing the nutritional status of children with CHD, including recent illness or insufficient dietary intake, feeding difficulties e.g., dysphagia, fatigue, gastroesophageal reflux disease (22), pulmonary hypertension (odds ratio [OR] ≈ 1.9), and congestive heart failure, which impede growth via chronic hypoxia, reduced perfusion, gut congestion, and nutrient malabsorption (10, 16). These findings indicate a considerable prevalence of malnutrition among children, highlighting the necessity for comprehensive nutritional evaluations and targeted interventions to enhance growth outcomes and overall health conditions, particularly in children with CHD within the first 1,000 days of life, and to prevent long-term impacts on physical and cognitive development (23, 24).

A crucial advantage lies in the prospective, multicenter design that encompasses three public hospitals catering to diverse service areas within Bannu District. This design enhances internal validity and local representativeness. All anthropometric measurements were standardized in accordance with the WHO Growth Standards, and multivariable modeling was rigorously adjusted for factors such as age, sex, locality, income, and maternal education.

Limitations include a cross-sectional design that cannot establish temporal relationships or causal inferences and a single-district setting that restricts national generalizability. Nevertheless, the demographic and healthcare characteristics of Bannu are generally representative of similar LMIC regions. It is recognized that recruitment from tertiary care hospitals may have resulted in a different sample than the broader population of CHD children. Additionally, higher socioeconomic groups are underrepresented, attributable to their preferential utilization of private healthcare facilities. However, this sampling bias inadvertently captures the population most susceptible to malnutrition - related morbidity. Some variables, including heart failure class, pulmonary hypertension, oxygen saturation, feeding difficulties, timing of surgical intervention, and recurrent infections, were not included in the analysis, which may have influenced the study findings.

## Conclusion

Children with CHD have high rates of undernutrition, including wasting and stunting, with several associated factors. These findings highlight the importance of integrating routine nutritional assessment at the time of CHD diagnosis, providing family-centered feeding counseling, and strengthening links between pediatric cardiac services, community growth monitoring, and nutrition support programs. Population based study and incorporation of standardized clinical measures like severity, heart failure status, oxygen saturation, pulmonary hypertension and feeding difficulties can play a vital role in providing representative findings and generalizability. Early identification and timely nutritional intervention may help prevent further nutritional deterioration and improve health outcomes among children with CHD. This includes expanding the implementation of antenatal echocardiography for early detection, subsidizing CHD medications and therapeutic foods within public health systems, according priority to nutritional rehabilitation for rural and male infants, and empowering caregivers through context-appropriate feeding education and growth monitoring. Addressing these intersecting biological and social determinants is crucial for enhancing the survival, growth, and long-term developmental outcomes of children with CHD in resource - limited settings.

## Data Availability

The original contributions presented in the study are included in the article/Supplementary material, further inquiries can be directed to the corresponding authors.
