# Supplementary material for: Addressing the burden and associated factors of undernutrition in under-five children with congenital heart disease in Pakistan: evidence from a multicenter cross-sectional study
Source: Front Nutr. 2026 Oct 16;13:1953133. doi: 10.3389/fnut.2026.1953133 (PMC13719390; doi:10.3389/fnut.2026.1953133)
Supplement: Supplementary file 1 [file Table_1.docx]

**Supplementary File**

| **Table S1 Clinical Characteristics of Children with CHD (n=197)** | | | |
| --- | --- | --- | --- |
| **Variable** | **Category** | **Frequency, n** | **Percentage (%)** |
| **Diagnosis (most severe lesion)** | Ventricular septal defect (VSD) | 68 | 35.1 |
|  | Atrial septal defect (ASD) | 42 | 21.7 |
|  | Patent ductus arteriosus (PDA) | 46 | 23.7 |
|  | Tetralogy of Fallot (TOF) | 28 | 14.4 |
|  | Transposition of the great arteries (TGA) | 10 | 5.1 |
| **First- or second-degree family history of CHD** | Yes | 8 | 4.1 |
|  | No | 189 | 95.9 |
| **Current cardiac pharmacotherapy** | Yes | 134 | 68.0 |
|  | No | 63 | 32.0 |
| **Prior corrective/palliative procedure** | Yes | 40 | 20.3 |
|  | No | 157 | 79.7 |
| **Routine nutritional supplementation** | None | 136 | 69.0 |
|  | Vitamin D drops | 7 | 3.6 |
|  | Calcium | 15 | 7.6 |
|  | Protein | 4 | 2.0 |
|  | Ready-to-use therapeutic food (RUTF | 7 | 3.6 |
|  | Vitamin B complex | 4 | 2.0 |
|  | Iron polymaltose | 10 | 5.1 |
|  | Folic acid | 1 | 0.5 |
|  | Yes, but not specified | 13 | 6.6 |
| **Immunization status (per Pakistan EPI schedule)** | Fully immunized | 149 | 75.6 |
|  | Partially immunized | 31 | 15.8 |
|  | Not immunized | 17 | 8.6 |
| **Primary caregiver with physician-diagnosed chronic illness** | Yes (including "anemia") | 2 | 1 |
|  | No | 195 | 99 |

**Table S2 Mean and the Standard Deviation of the Anthropometric Measurements of Children with CHD**

| **Variable** | **Mean ±SD** | **95% CI** |
| --- | --- | --- |
| Weight (kg) | 9.17 ± 3.34 | 8.71–9.64 |
| Length/Height (cm) | 78.02 ± 14.32 | 76.03–80.02 |
| Mid Upper Arm  Circumference (MUAC, cm) | 12.01 ± 1.89 | 11.74–12.27 |

**Table S3 Mean WHO growth standard–based Z-scores by age group and gender among children with congenital heart disease**

|  | **Indicator** | **Mean Z-score** | **95% Confidence Interval** |
| --- | --- | --- | --- |
| Overall | HAZ | -0.82 | -1.95-1.69 |
| Overall | WAZ | -1.84 | -1.98 -1.71 |
| Overall | WHZ | -1.09 | -1.23-0.95 |
| Age 6–11 months | HAZ | -2.08 | -2.26-1.90 |
| Age 6–11 months | WAZ | -2.46 | -2.67 -2.25 |
| Age 6–11 months | WHZ | -1.21 | -1.38-1.04 |
| Age 12–24 months | HAZ | -1.89 | -2.05-1.73 |
| Age 12–24 months | WAZ | -1.92 | -2.10-1.74 |
| Age 12–24 months | WHZ | -1.15 | -1.32 -0.98 |
| Boys | HAZ | -1.93 | -2.10-1.76 |
| Boys | WAZ | -1.88 | -2.05 to-1.71 |
| Boys | WHZ | -1.05 | -1.22-0.88 |
| Girls | HAZ | -1.71 | -1.88 to-1.54 |
| Girls | WAZ | -1.80 | -1.97 -1.63 |
| Girls | WHZ | -1.14 | -1.31-0.97 |

**Figure S1 Nutritional Status of Children with CHD**
